# Biological Insights From the Osteohistology of a Nile Crocodile

**DOI:** 10.1002/jmor.70186

**Published:** 2026-10-26

**Authors:** Ayanda Buso, Maria Eugenia Pereyra, Anusuya Chinsamy

**Affiliations:** ^1^ Department of Biological Sciences University of Cape Town Cape Town South Africa

**Keywords:** crocodilian, osteoderms, skeletochronology

## Abstract

While long bones are mostly utlised for skeletochronology of extant and extinct vertebrates, among crocodilians, dermal bones have also been studied. However, there is a lack of a systematic and comprehensive study of the variation in osteohistology and skeletochronology of limb, axial, and dermal bones of extant crocodilians. Here, we undertake a comprehensive assessment of the histology of multiple postcranial elements of a single, captive adult *Crocodylus niloticus* (with approximately known age) to assess their utility for skeletochronology. We document the intraskeletal histological variability in the skeleton and show that despite the gastralia recording the highest number of growth rings, the radius, ulna, and fibula are more reliable for skeletochronology. We show substantial variation in the histology of the osteoderms depending on the plane of sectioning, as well as their location on the body of the crocodile. Additionally, we report that anteriorly sectioned dorsal osteoderms preserved the highest number of growth marks. Our study provides important information regarding intraskeletal variation in osteohistology and presents key information regarding the choice of skeletal elements for reliable life history reconstruction of *C. niloticus*.

## Graphical abstract

Intraskeletal histological analysis of *Crocodylus niloticus* shows substantial variation in growth mark preservation across postcranial elements. Gastralia preserve the highest number of growth marks, while the radius, ulna, and fibula are the most reliable long bones for skeletochronology. The anterior dorsal osteoderm matches these long bones in growth mark count, pointing to a minimally invasive alternative for age estimation.

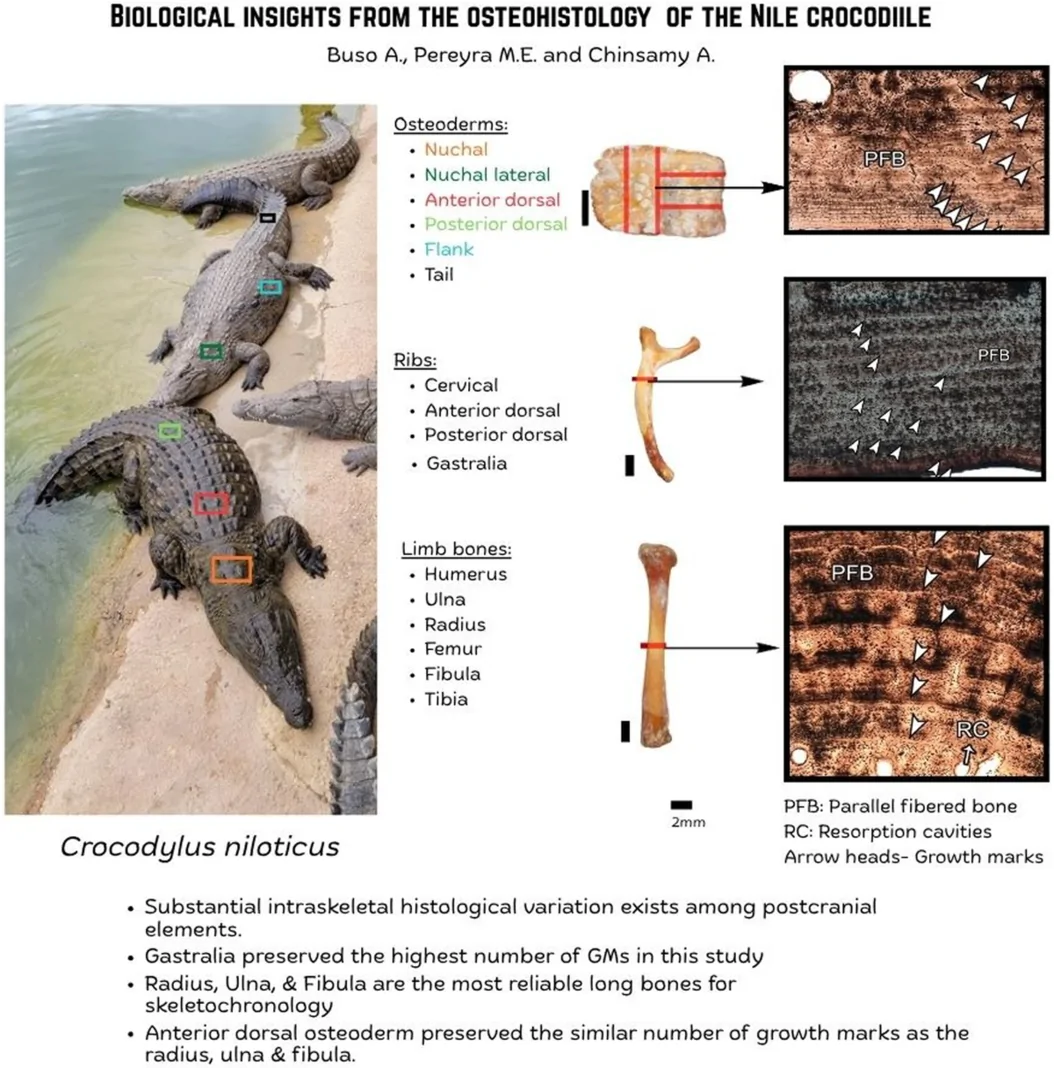

**Abbreviations**
CCCB: compacted coarse cancellous bone
GMs: growth marks
ICL: inner circumferential layer
LAGs: lines of arrested growth
MC: medullary cavity
MR: medullary region
PFB: parallel‐fibered bone
PO: primary osteon
RL: resorption line
RCs: resorption cavities
SF: Sharpey's fibers
SO: secondary osteons
TB: trabecular bone
WPC: woven parallel complex

## 1 Introduction

Osteohistology permits a reconstruction of various aspects of the biology of an animal, such as growth rate, physiology, diseases, sexual maturity, among others (e.g., Chinsamy‐Turan 2005; Chinsamy 2023; Erickson 2005; Sander et al. 2004). Growth marks (GMs), such as, annuli and lines of arrested growth (LAGs), are formed within the bone cortex (e.g., Castanet 1994; Francillon‐Vieillot et al. 1990). LAGs represent cessation of bone deposition, while the annuli are narrow layers that represent periods of slower depositional rates and typically consist of parallel‐fibered bone (PFB) or lamellar bone (LB). Skeletochronology is a method used to estimate the age of vertebrates by counting the number of GMs preserved in their bone tissues (e.g., Caetano and Castanet 1993; Castanet 2006; Chinsamy‐Turan 2005; Peabody 1961). Midshaft sections of stylopodial bones are generally considered the most reliable skeletal elements for skeletochronology, however, the use of other postcranial elements, such as ribs and osteoderms, have also been used (e.g., Caetano and Castanet 1993; Castanet and Baez 1991; Cerda et al. 2022; Ponce et al. 2021; Snover and Rhodin 2007). The most reliable skeletal element for age estimation varies in different vertebrates (e.g., ribs of sauropod dinosaurs, Waskow and Mateus 2017; tibia of the tortoise, *Chersina angulata*, Bhat et al. 2019; fibula of the turtle, *Phrynops hilary*, Pereyra 2023). Among modern crocodilians, the fibula preserves the best record of growth marks in *Crocodylus siamensis* (de Buffrénil 1980), the radius in *Alligator mississippiensis* (Woodward et al. 2014), while it is the fibula, ulna, and radius in *Caiman latirostris* (Pereyra et al. 2024).

The bone histology of modern crocodilians has been well studied to infer biological information (e.g., Audije‐Gil et al. 2023; Chinsamy 1991; de Buffrénil 1980; Pereyra et al. 2024; Pereyra et al. 2025; Woodward et al. 2014) and such studies have often been extrapolated to extinct crocodylomorphs and other archosaurs (e.g., Cerda et al. 2018; Cerda et al. 2022; Navarro et al. 2023; Ponce et al. 2026; Teschner et al. 2022). Although GMs are generally considered annual, several studies have shown that this is not necessarily always the case (e.g., Chinsamy and Pereyra 2026; Pereyra et al. 2024; Schucht et al. 2021).

Several osteohistological studies have comprehensively analysed multiple osteoderms from different body regions of single individuals. However, majority of these are on stem pseudosuchians or other archosauriform taxa (e.g., Błaszczeć and Antczak 2025; Cerda et al. 2018; Cerda et al. 2013; Scheyer and Desojo 2011). Additionally, most have focussed on sections oriented perpendicular to the keel (e.g., Clarac et al. 2018; Hill 2010; Navarro et al. 2024; Scheyer and Desojo 2011), whereas some have explored multiple sectioning planes (e.g., Burns et al. 2013; Cerda and Desojo 2011; Cerda et al. 2015; Tucker 1997). A few studies have investigated the histology of osteoderms of *Crocodylus niloticus* (Clarac et al. 2019; de Buffrénil et al. 2015; Games 1990; Hutton 1986). However, in the current study, we adopt an integrative, multi‐regional and multi‐sectional approach. Here, we undertake a comparative osteohistological analysis of postcranial bones and osteoderms from various parts of the body of a captive, *C. niloticus* which enabled us to assess intraskeletal histo‐variation, and to determine which elements maintained the best growth record. Additionally, the results of this study will allow extrapolation to the fossil record of archosaurs and will permit more reliable choices of skeletal elements for palaeobiology and skeletochronology assessments.

## 2 Materials and Methods

The approximately 14‐year‐old crocodile studied here, of unknown sex, died of natural causes and was donated to us by Le Bonheur Reptile Adventure‐Croc and Reptile Park, located in Western Cape Winelands in Paarl. The partially decomposed specimen was skeletonized at the University of Cape Town's Medical School, and several limb, axial and dermal bones were selected for histological analysis (Tables S1–S3). Osteoderms from six parts of the body were chosen for study: nuchal, nuchal lateral, dorsal‐anterior, dorsal‐posterior, flank, and tail. All the bones were measured, photographed (Tables: S1–S3; Figure S1–S3) and CT scanned (by X‐sight X‐Ray services in Somerset West) and analysed using the software myVGL (2024.4., version: 0.2.1.0) (Figures S1–S3). Prior to sectioning, the bones were dehydrated and defatted following Pereyra et al. (2024) protocol. Thereafter, the limb bones were sectioned at the midshaft, and the ribs were sectioned at the proximal region. The osteoderms were sectioned in four planes: two antero‐posterior sections (“over the keel” and “parallel to the keel”), and two latero‐medial sections [“transverse to the keel (anterior)” and “transverse to the keel (posterior)”] (Figures S1–S3).

The skeletal elements were thin sectioned using the standard petrographic protocol (Cerda et al. 2020; Chinsamy and Raath 1992) and were studied under a Zeiss petrographic microscope with a camera (Axiocam 208) at the University of Cape Town. The images were processed using Photoshop and Autopano Giga. The terminology follows Francillon‐Vieillot et al. (1990), Chinsamy‐Turan (2005), Prondvai et al. (2014) and de Buffrénil and Quilhac (2021). For osteoderms, following Cerda et al. (2015) we use the terms “external cortex”, “basal cortex”, and “internal core”. The growth marks (GMs) in the limb bones were counted from the perimedullary region to the periosteal surface, and in the osteoderms were counted from the inner core to the surface of the external and basal cortices.

## 3 Results

Below, we provide a summarized description of the histology of each element. More detailed information of each of the 33 thin sections prepared is provided in the supplementary Table S4.

### 3.1 Axial and Appendicular Bones

Note that when GMs are present, they tend to be closer together nearer the periosteal surface.

#### 3.1.1 Humerus

The humerus consists of an open MC surrounded by a thick cortex (Figure 1A). The cortical bone is predominantly composed of PFB, with some WPC present in the inner cortex of the anterior regions of the section (Figure 1B,C). There are simple canals and primary osteons with longitudinal, radial, and reticular orientation. The vascularization density decreases towards the outer surface of the bone. RCs occur in the inner and middle cortex and are more abundant in the posterior area (Figure 1B). SFs are especially abundant in the posterior and lateral area of the cortical bone. 11 annuli with LAGs were recorded.

**Figure 1:**
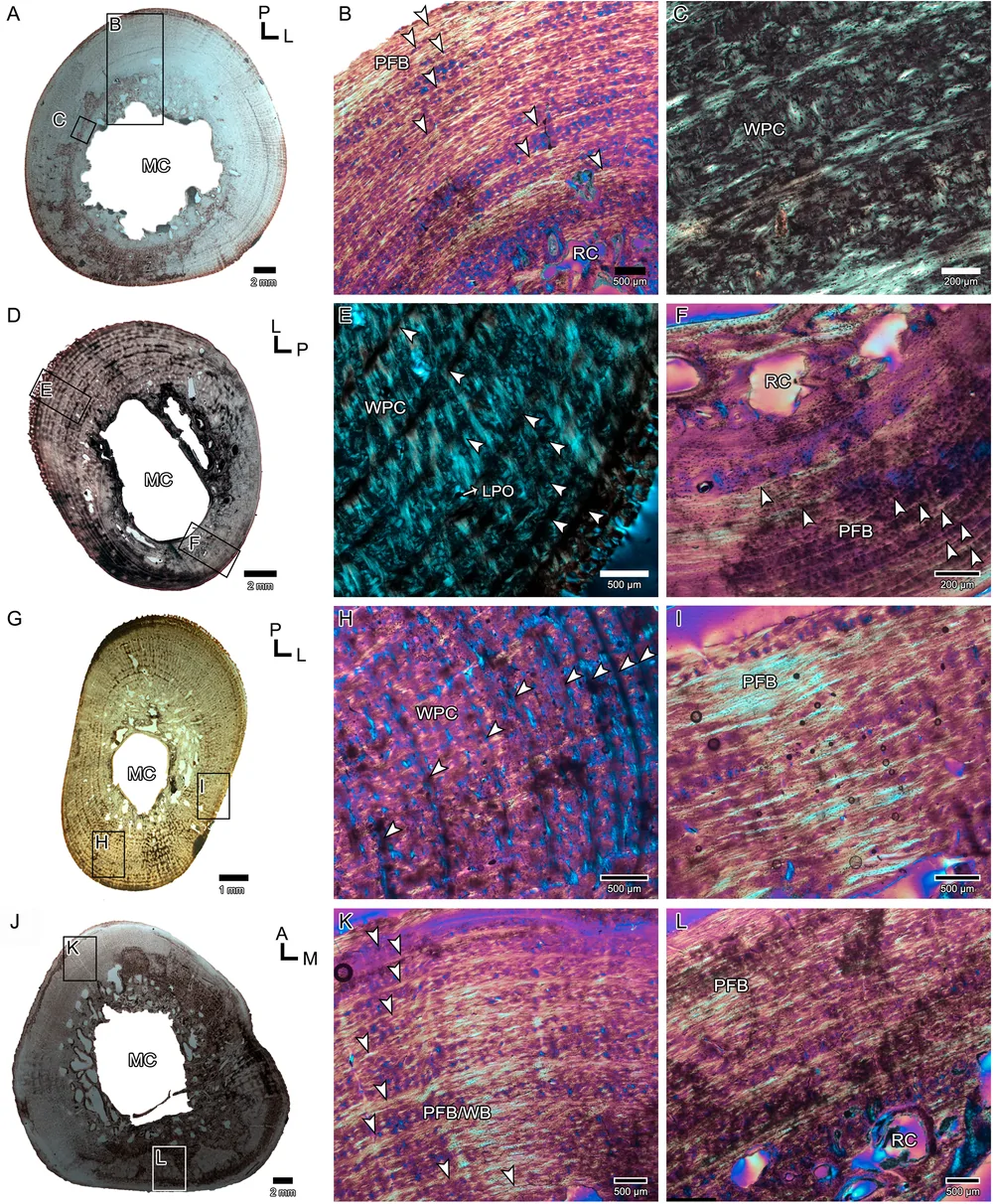
Thin sections of appendicular bones of *C. niloticus*. (A) overview of the humerus cross section. Frames indicate location of the higher magnification images in (B) and (C), (B) higher magnification of the posterior area of the humerus, showing the bone tissue types and GMs (arrowheads), (C) Inner cortex of the medial area, showing the WPC bone tissue, (D) Overview of the radius cross section. Frames indicate location of the higher magnification images in (E) and (F), (E) outer cortex of the radius, showing the WPC with LPO in the zones, arrowheads indicate GMs, (F) medial area of the radius showing PFB and GMs (arrowheads), as well as resorption cavities in the perimedullary region, (G) overview of the ulna cross section. Frames indicate location of the higher magnification images in (H) and (I), (H) anterior area showing the zones comprising of WPC and GMs (arrowheads); (I) outer cortex of the lateral area of the ulna, showing PFB, (J) overview of cross section of the femur. Frames indicate location of the higher magnification images in (K) and (L), (K) anterior area of the femur showing, WPC and PFB, (L) posterior area of the femur showing predominantly PFB with some RCs in the perimedullary region. A, anterior area; L, lateral area, LPO, longitudinal primary osteon; MC, medullary cavity; M, medial area; PFB, parallel‐fibered bone; P, posterior area; RC, resorption cavity; WPC, woven parallel‐complex; A,D, G, J, normal light; B, F, H‐L, cross‐polarized with ¼ lambda filter; C, E, cross‐polarized light.

#### 3.1.2 Radius

The radius consists of an open MC surrounded by an unevenly thick bone wall (with the thickest side on the lateral side). RCs and SO are present in the inner and mid cortex. The cortical bone consists of zonal bone of WPC and PFB at the lateral area, while the medial area consists of PFB (Figure 1D–F). The vascularization consists of simple canals and primary osteons with longitudinal and radial orientations, except for the medial area which is almost avascular. There are SFs in the posterior and anterior areas which have an oblique orientation (medial to lateral direction). 14 LAGs were recorded.

#### 3.1.3 Ulna

An open MC is surrounded by a thick bone wall with several large RCs in the inner and mid cortex, and a few small ones located in the outer cortex (Figure 1G). Here, WPC occurs in the inner and mid parts of the posterior and anterior areas, whereas the PFB dominates the outer cortex and the whole lateral area (Figure 1H,I). Abundant simple canals and PO with different orientations (radial, longitudinal, reticular) are observed. SFs with variable orientations occur especially in the posterior area of the bone wall. The ulna has 14 LAGs in the compacta.

#### 3.1.4 Femur

A compacta surrounds a large open MC (Figure 1J). The cortical bone consists of PFB with woven texture, with more organized PFB in the outer cortex (Figure 1K). Overall, the bone is well vascularised (simple canals and primary osteons), although this density decreases in the outer cortex. A large number of RCs occur in the inner and mid parts of the cortex (Figure 1L). The SFs are oriented perpendicular to the periosteal surface. A total of 11 annuli with LAGs were recorded with a decreasing distance between them towards the bone surface.

#### 3.1.5 Fibula

An open MC is surrounded by a thick compact bone wall, which consists of zonal bone with WPC and PFB, while the outer cortex consists of PFB with LAGs (Figure 2A–C). Vascularization (simple canals and primary osteons) tends to decrease from the inner to the outer regions of the bone wall (Figure 2B,C). Although the main orientation of the canals is longitudinal, there are also radial, reticular, and circumferential canals. RCs occur in the inner and mid cortex but reach the outer cortex of the anterior area. SFs occur in the middle and outer cortex especially on the postero‐medial and postero‐lateral area and occur perpendicular to the surface. A total of 14 LAGs were recorded, with the outer ones being closer together.

**Figure 2:**
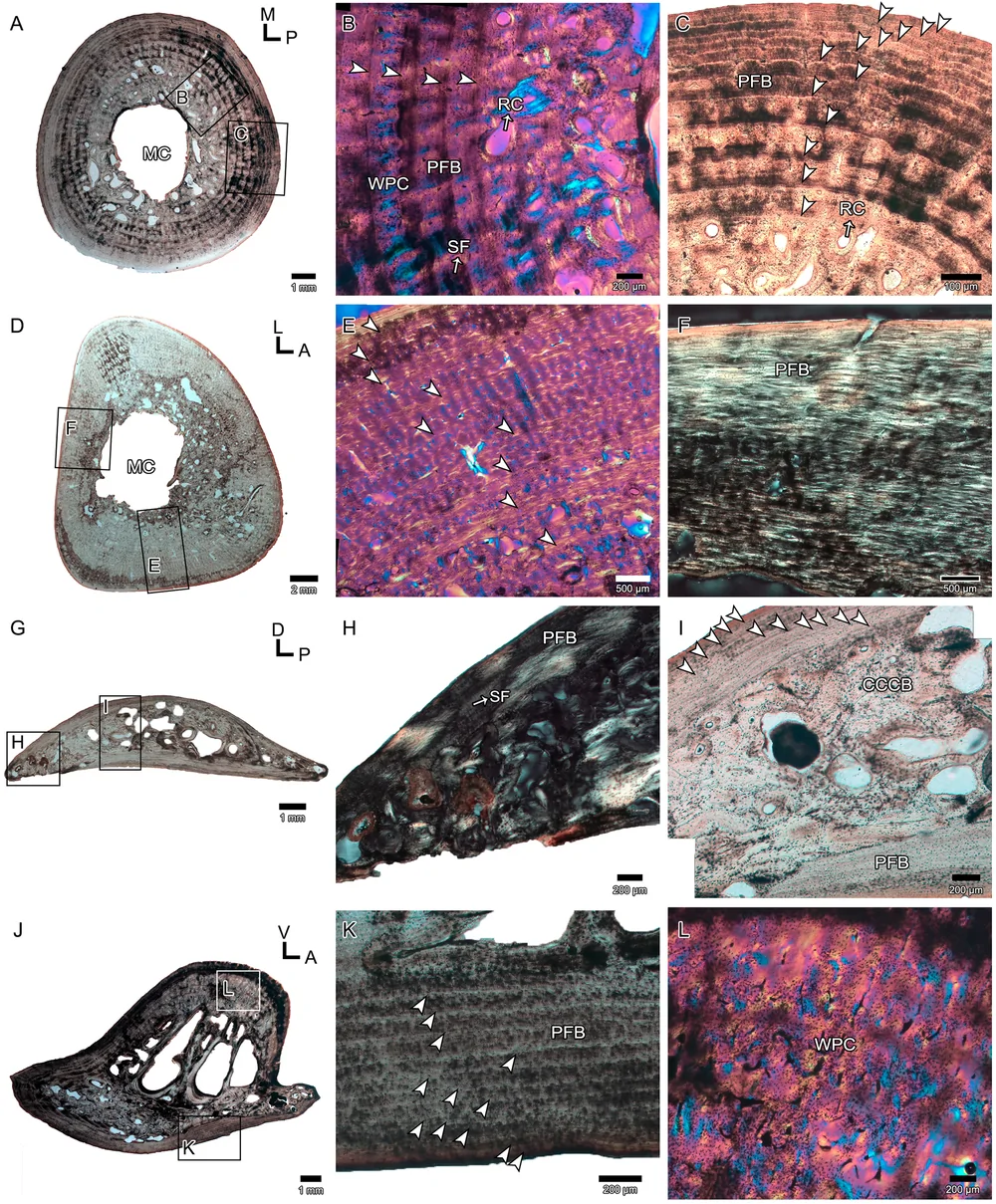
Thin sections of axial and appendicular bones of *C. niloticus*. (A) Overview of the fibula cross section. Frames indicate location of the higher magnification images in (B) and (C), (B) inner cortex of the posteromedial area of the fibula showing PFB, WPC in the zones. Arrowheads indicated GMs, (C) posterior area of the fibula showing the general PFB nature of the compacta and GMs (arrowheads) (D) overview of the tibia cross section. Frames indicate location of the higher magnification images in (E) and (F), (E) medial area of the tibia showing GMs (arrowheads), (F) outer cortex of the tibia, showing the PFB dominating the outer parts of the compacta, (G) overview of the cross section of the cervical rib. Frames indicate location of the higher magnification images in (H and (I), (H) anterior area of the rib showing PFB and SFs, (I) central area of the rib showing the PFB interrupted by GMs (arrowheads) and the widespread occurrence of CCCB centrally, (J) overview of the cross section of the dorsal rib (anterior). Frames indicate location of the higher magnification images in (K) and (L), (K) dorsal area of the rib showing avascular PFB with LAGs (arrowheads), (L) ventral area of the rib showing WPC. Abbreviations not mentioned in Figure 1D: dorsal area, SF: Sharpey's fibers, V: ventral area. A, C, D, G, I, J, K: normal light, B, E, L: cross polarized with ¼ lambda filter, F, H: cross polarized light.

#### 3.1.6 Tibia

The tibia consists of an open MC surrounded by an unevenly thick compacta, which is particularly narrow in the posterior part of the bone (Figure 2D). Overall, the primary bone tissue at the inner and mid parts comprises WPC, while PFB predominantly occurs in the outer cortex (Figure 2E,F). This section is well vascularised by simple canals and primary osteons. RCs and SO tend to occur in the inner cortex. There are abundant SFs at the lateral area with a perpendicular orientation to the surface of the bone. A total of 13 annuli with LAGs were recorded.

#### 3.1.7 Cervical Rib

No clear medullary cavity is present. A thin compacta with RCs and CCCB in the anterior and posterior areas of the inner cortex and parts of the middle cortex is present (Figure 2G,H). The primary bone tissue consists of PFB (Figure 2H,I). The cortex is almost avascular with a few longitudinal canals occurring in the posterior area of the inner and middle cortex. There is a high density of SFs obliquely oriented in the ventral and dorsal areas. A total of 11 closely spaced LAGs occur (Figure 2I).

#### 3.1.8 Dorsal‐Anterior Rib

The medullary region of the rib is filled with trabeculae bone and is surrounded by a layer of unevenly thick compact bone (Figure 2J). The cortical bone consists mainly of avascular PFB at the dorsal area and WPC at the ventral area of the section (Figure 2K,L). The vascularization is more abundant in the middle cortex of the anterior and posterior areas and in the whole thickness of the ventral area, with canals arranged longitudinally and showing occasional reticular anastomoses. RCs are abundant in the inner and middle cortices. The SFs are present in the anterior and posterior areas. There are 10‐12 closely associated LAGs.

#### 3.1.9 Dorsal Posterior Rib

Predominantly PFB occurs in the inner, middle and outer regions of the bone wall (Figure 3A–C). The dorsal area of the outer cortex is vascularized (longitudinal canals), while the rest of the cortex is avascular. The middle cortex consists of isolated RCs which are lined with lamellar bone. A thick ICL surrounds the MC and shows 4 RL (Figure 3B). There are SFs in the dorsal and posterior areas. A total of 11 LAGs were recorded, and some of the outer ones appear to be double (Figure 3C).

**Figure 3:**
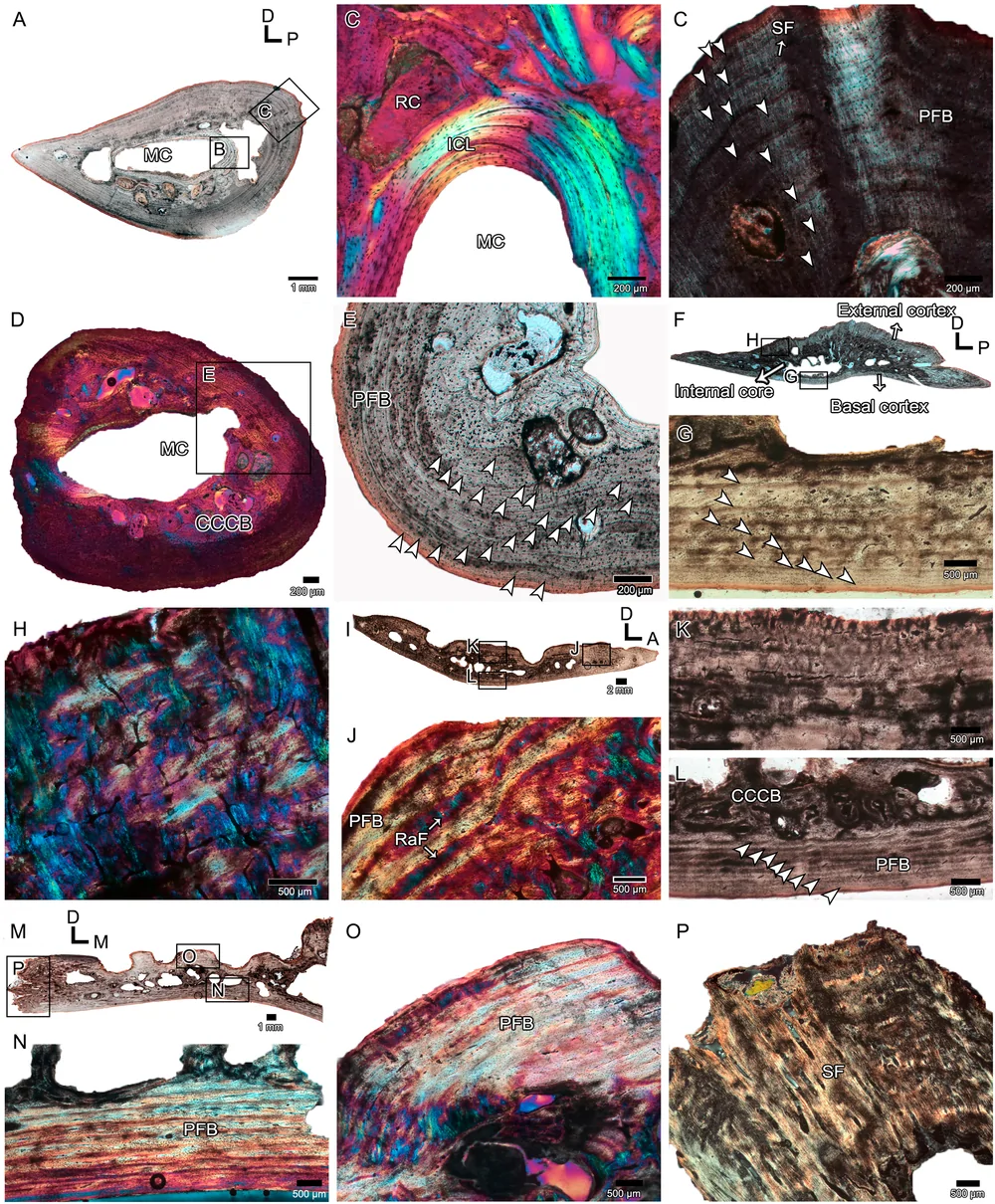
Thin sections of axial bones and osteoderms of *C. niloticus*. (A) Overview of the cross section of the dorsal rib (posterior). Frames indicate location of the higher magnification images in (C) and (B), (B) posterior area of the rib showing the ICL with several rest lines lining the medullary cavity, (C) posterior area of the rib showing the PFB interrupted by GMs (arrowheads), (D) overview of the cross section of the gastralia. Frame indicates location of the higher magnification image in (E), (E) outer cortex of the gastralia showing the densely packed GMs (arrowheads), (F) overview of the cross section “over the keel” of the nuchal osteoderm. Frame indicates location of the higher magnification images in (G) and (H), (G) basal cortex showing the GMs (arrowheads), (H) external cortex showing WPC with reticular organized canals, (I) overview of the “parallel to the keel” cross section of the nuchal lateral osteoderm. Frames indicate the location of the higher magnification images in (J), (K), and (L), (J) and (K) show the nature of the external cortex of the osteoderm; (L) basal cortex showing growth marks (arrowheads) and CCCB, (M) overview of the “transverse to the keel (anterior)” cross section of the nuchal osteoderm. Frames indicate the location of the higher magnification images in (N), (O) and (P), (N) basal cortex and (O) external cortex of the osteoderm showing PFB interrupted by GMs (arrowheads), (P) lateral end of the osteoderm showing the distribution of SFs. Abbreviation not mentioned in Figures 1 and 2: RaF: radial fibers, A, E, F, G, I, K ‐ M: normal light, B, D, H, J, N, O: cross polarized with 1/4 lambda filter, C, P: cross polarized light.

#### 3.1.10 Gastralia

The gastralia consists of an open MC surrounded by a thick bone wall (Figure 3D), comprising predominantly of PFB (Figure 3E) with few simple canals. There is large amount of secondary reconstruction with RCs, SO and CCCB occurring in the inner cortex. The outer cortex is almost avascular. There are SFs in the anterior and medial area of the middle and outer cortex. A total of 24 LAGs was recorded, some in groups of two and three.

### 3.2 Osteoderms

Six osteoderms from different parts of the crocodile were sectioned in different planes. Detailed descriptions are in the Supporting material Table S4. Here we provide a summary of the osteohistology of the osteoderms. All the osteoderms, have a highly variable cortical thickness, with the “over the keel” section having the thickest external cortex. The dorsal surfaces tend to be uneven and irregular due to the ornamentation, while the ventral surfaces are smooth.

#### 3.2.1 Nuchal

In general, the cortices are mainly composed of PFB with localized WPC while the internal core comprises of CCCB with patches of WB, large RCs and SO (Figures 3F–P, 4A–C). Some regions of the external cortex present vertical fibers (i.e., transverse with respect to the dorsal surface of the osteoderm) interspersed with longitudinal fibers (Figure 3J). The bone is vascularised by mostly longitudinal canals in the basal cortex; while some reticular canals occur in the external cortex (Figures 3, 4). Compared with the external cortex of the “over the keel” section (Figure 3F–H), the “parallel to the keel” (Figure 3I–L) and “transverse to the keel” (Figures 3M–P, 4A–C) sections are much more scalloped with irregular ridges and valleys. The highest number of GMs (12) were observed in the basal cortex of the “over the keel” section, while other sections showed fewer LAGs, the least (8) recorded in the basal cortex of the “transverse to the keel (posterior)” sections.

**Figure 4:**
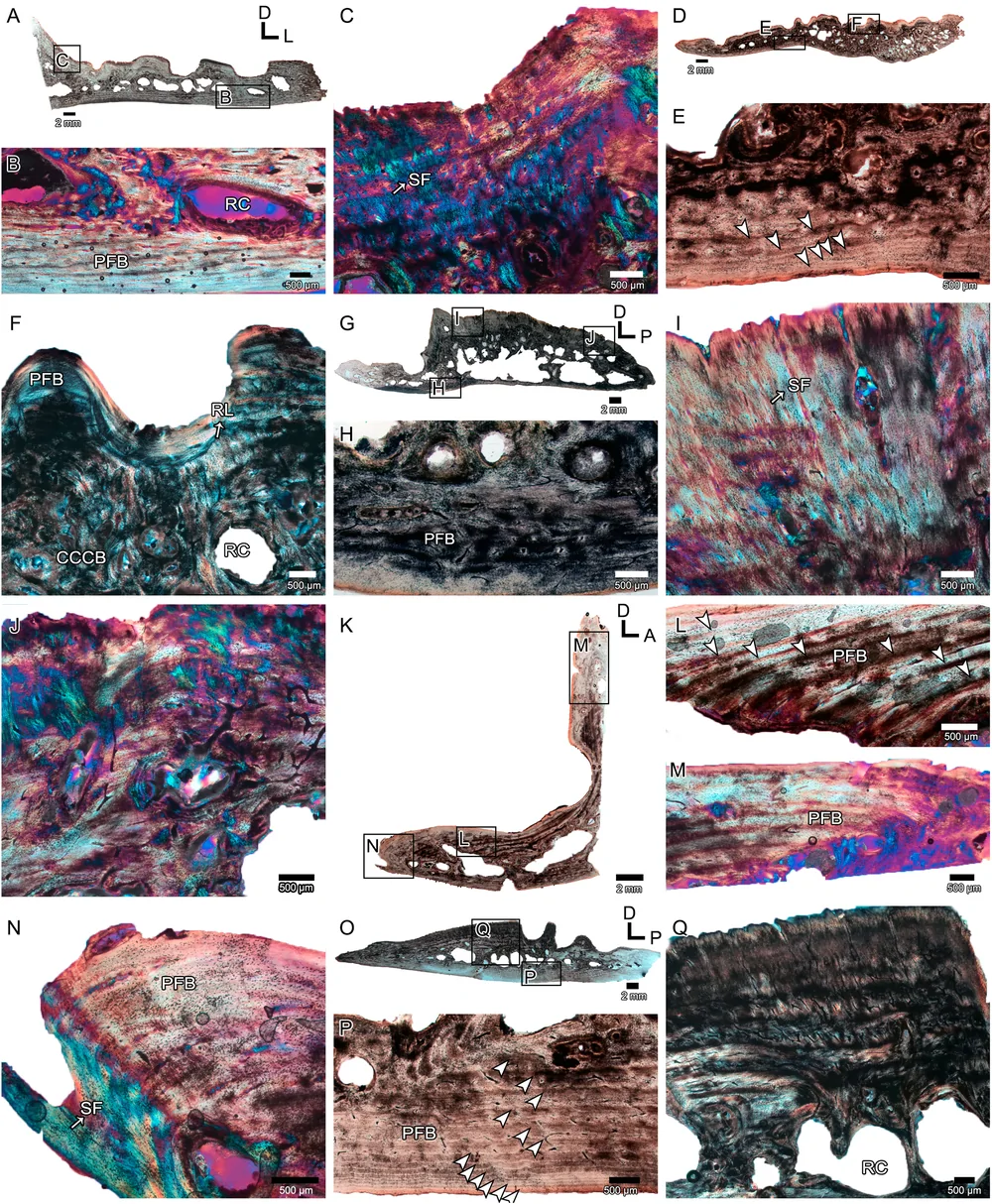
Thin sections of osteoderms of *C. niloticus*. (A) Overview of the “transverse to the keel (posterior)” cross section of the nuchal osteoderm. Frames indicate location of the higher magnification images in (B) and (C), (B) basal cortex showing PFB, (C) external cortex showing high density of SFs and the general WPC bone tissue; (D) overview of the “parallel to the keel” cross section of the nuchal lateral osteoderm. Frames indicate the location of the higher magnification images in (E) and (F), (E) basal cortex showing GMs (arrowheads), (F) external cortex, showing PFB, (G) overview of the “over the keel” cross section of the nuchal lateral osteoderm. Frames indicate the location of the higher magnification images in (H), (I) and (J), (H) basal cortex showing the PFB, (I) and (J) external cortex showing SFs, (K) overview of the transverse section of the nuchal‐lateral osteoderm. Frames indicate the location of the higher magnification images in (L), (M) and (N), (L) and (M) external cortex showing PFB with GMs (arrowheads), (N) lateral end of the osteoderm with similar tissues and SFs, (O) overview of the “over the keel” cross section of the anterior dorsal osteoderm. Frames indicate the location of the higher magnification images in (P) and (Q), (P) basal cortex showing PFB with GMs (arrowheads), (Q) external cortex showing WPC. Abbreviations not mentioned in Figures 1 and 2: RL: resorption line. A, D, E, G, K, O, P: normal light, B, C, I, J, M, N: cross polarized with ¼ lambda filter, F, Q: cross polarized light.

#### 3.2.2 Nuchal Lateral

The cortices are mainly composed of PFB with some regions with WPC; however, the basal cortex tend to be more organized. In some regions, the abundance of SFs do not allow the proper definition of the primary bone tissue. The CCCB make up the internal core, and RCs and SO also occur here (Figure 4D–N). Simple canals and PO occur with longitudinal, radial and reticular orientations, although the basal cortex is less vascularized than the external cortex. SFs are particularly abundant in the external cortex as well as at the medial side of the osteoderms (Figure 4I,N). 13 LAGs were observed in the external cortex of the “over the keel” section, whereas the basal cortices preserved between 8 and 10 LAGs (Table S4).

#### 3.2.3 Dorsal‐Anterior

The cortices are mainly composed of PFB and WPC (Figures 4, 5). While the sections “over the keel” and “parallel to the keel” show more WPC, the sections “transverse to the keel” show a predominance of PFB. The internal core is mainly composed of CCCB, with abundant RCs and SO (Figures 4, 5). Vascular canals with different orientations are abundant in the external cortex, while the basal cortex consists mainly of longitudinal canals. SFs are abundant in the external cortex in the sections “over the keel” and “parallel to the keel”, while they are abundant at the lateral end of the osteoderm in the sections “transversal to the keel” (Figures 4, 5). The basal cortex of the “over the keel” section preserved the highest number of LAGs (16), with all other regions having fewer LAGs, with as little as 11 in the external cortex of the “transverse to the keel (anterior)” section (Figure 5L,N).

**Figure 5:**
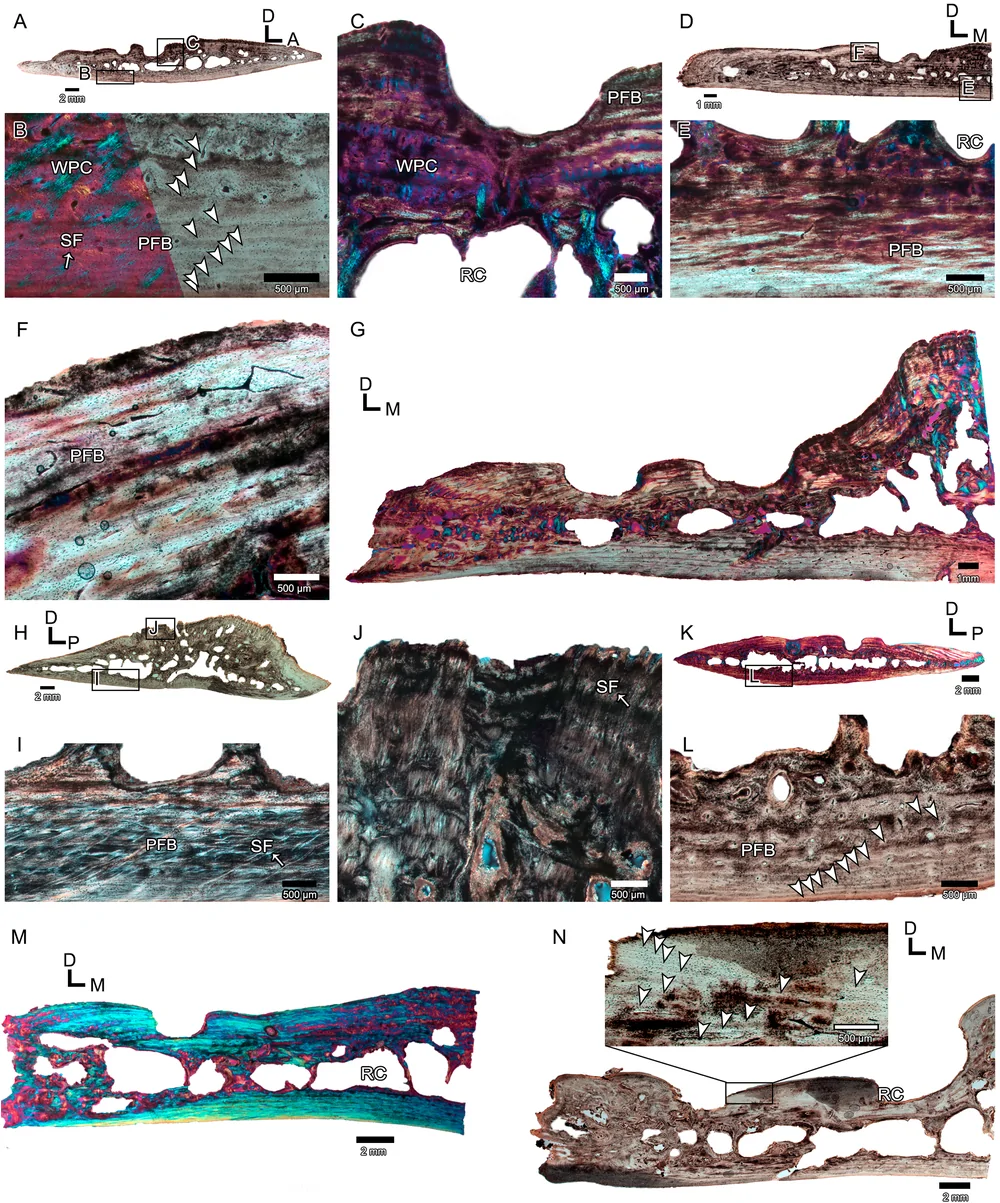
Thin sections of osteoderms of *C. niloticus*. (A) overview of the “parallel to the keel” cross section of the anterior dorsal osteoderm. Frames indicate the location of the higher magnification images in (B) and (C), (B) under polarized (left) and normal (right) light showing the SFs and GMs (arrowheads), (C) external cortex, showing PFB at the outermost part of the osteoderm and WPC at the inner region, (D) overview of the “transvers to the keel (anterior)” cross section of the anterior dorsal osteoderm. Frames indicate the location of the higher magnification images in (E) and (F), (E) basal cortex and (F) external cortex showing PFB, (G) overview of the “transverse to the keel (posterior)” cross section of the anterior dorsal osteoderm showing the trilaminar organization of the osteoderm with a highly porous internal core; (H) overview of “over the keel” cross section. Frames indicate the location of the higher magnification images in (I) and (J), (I) basal cortex showing PFB and SFs, (J) external cortex showing abundant SFs, (K) overview of “parallel to the keel” cross section of the posterior dorsal osteoderm. Frame indicates the location of the higher magnification image in (L), (L) basal cortex showing GMs (arrowheads), (M) overview of “transverse to the keel (anterior)” cross section of the posterior dorsal osteoderm, showing the highly porous internal core, particularly at the level of the keel, (N) overview of “transverse to the keel (posterior)” cross section of the posterior dorsal osteoderm, showing the ornamentation of the external cortex and the porosity of the internal core. Abbreviations see Figures 1 and 2 A, D, H, L, N: natural light, C, E, F, G, K, M: cross polarized light with ¼ lambda filter, I, J: cross polarized light, B: natural light + cross polarized light with ¼ lambda filter.

#### 3.2.4 Dorsal Posterior

In the “over the keel” section has almost no evidence of ornamentation, whereas in the other sections there is much more ornamentation (Figure 5H–N). In all the sections, the internal core is mainly composed of CCCB with large RCs (Figure 5M,N) and some extending to the cortices (Figure 5I). The basal cortex is generally composed of PFB while the external cortex presents PFB and WPC. However, the sections “transverse to the keel” have more abundant PFB (Figure 5I,J,L–N). The simple canals and primary osteons are frequent and present different orientations. SFs are present in different directions and are more abundant in the external cortex of the “over the keel” section (Figure 5J). The external cortex of the “parallel to the keel” section preserves 13 LAGs, while the lowest LAGs (10) were present in several sections such as “transverse to the keel (anterior)”.

#### 3.2.5 Tail

The “over the keel” section has thicker external cortex and an extremely thin basal cortex sandwiching an internal core with large RCs (Figure 6A–E). The different regions are composed of vascularized PFB with localized WPC. The internal core presents CCCB with RCs (Figure 6E). SFs occur abundantly in all the external cortex of the different sections. The basal cortex of the “parallel to the keel” and “transverse to the keel (anterior)” sections record the lowest number of LAGs (3), while 8–9 GMs are recorded in the external cortex of most of the other sections.

**Figure 6:**
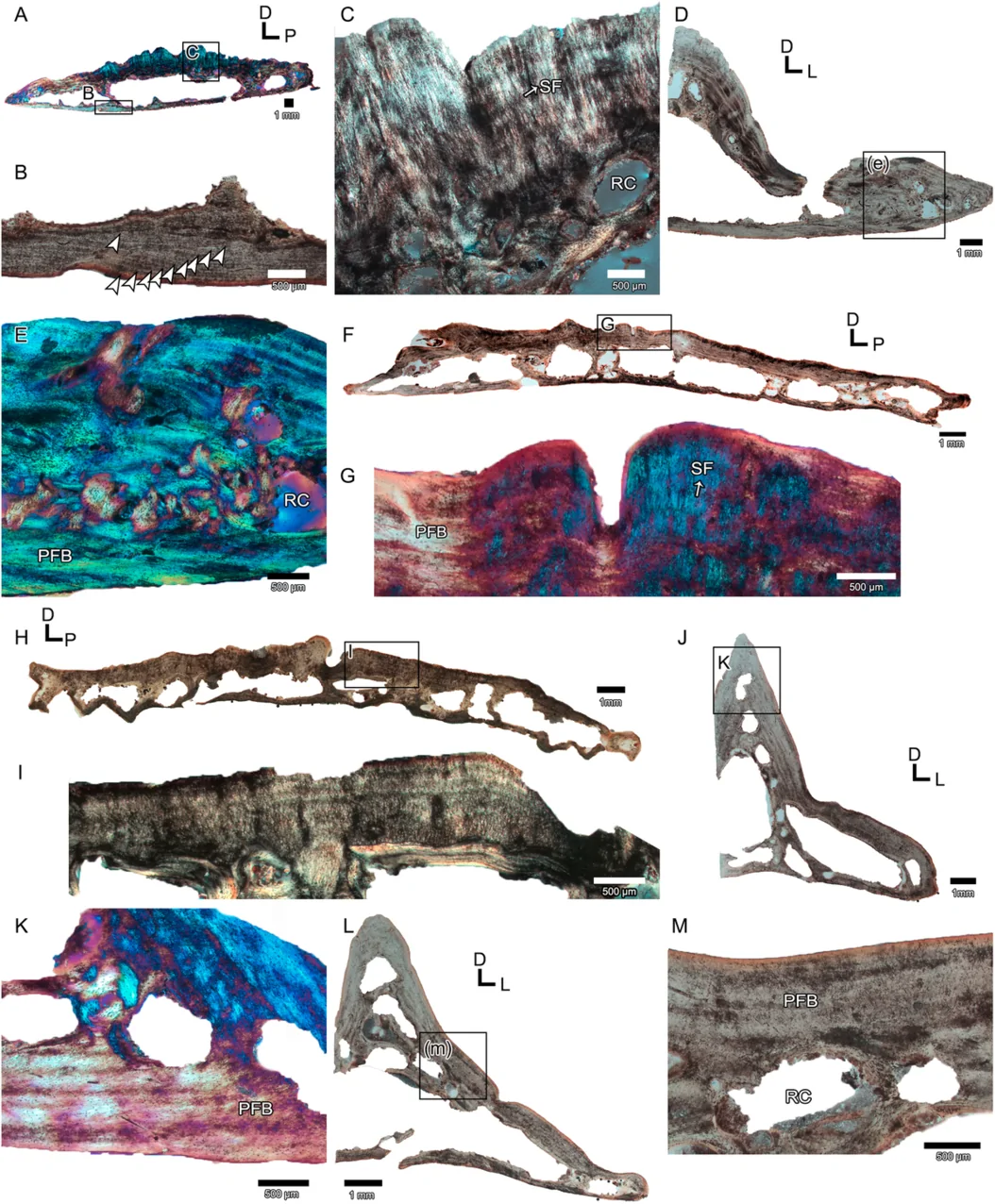
Thin sections of osteoderms of *C. niloticus*. (A) overview of the “over the keel” cross section of the flank osteoderm. Frames indicate the location of the higher magnification images in (B) and (C), (B) basal cortex showing the GMs (arrowheads), (C) external cortex showing SFs, (D) overview of the “transverse to the keel (anterior)” cross section of the flank osteoderm. The frame indicates the location of (E), (E) lateral end of the osteoderm showing the PFB and RCs, (F) overview of the “over the keel” section of the tail osteoderm. The frame indicates the location of (G), (G) external cortex showing the PFB and SFs; (H) overview of the “parallel to the keel” cross section of the tail osteoderm. The frame indicates the location of (I), (I) external cortex showing the SFs, (J) overview of the “transverse to the keel (anterior)” section of the tail osteoderm. The frame indicates the location of (K), (K) close up of the keel showing PFB in the external and basal cortices; (L) overview of the “transverse to the keel (posterior)” section of the tail osteoderm. The frame indicates the location of (M); (M) external cortex showing PFB and large RCs. Abbreviation see Figures 1 and 2 B, D, F, D, H, J, L, M: normal light, A, E, G, K: cross polarized with ¼ lambda filter, C, I: cross polarized light.

#### 3.2.6 Flank

The surface of the external cortex, especially on the “transverse to the keel” section is thicker (Figure 6F–M) and tends to have an irregular surface with shallow ornamentation, as compared to the ventral surface. The cortices are mainly composed of PFB (Figure 6G,I,K,M). Simple canals with different orientations predominate in the cortices. SFs are abundant in the external and basal cortices of most sections, although the basal cortex of the “parallel to the keel” section seems to have more in the basal cortex. 10 LAGs are present in the “transverse to the keel (posterior)” section, whilst fewer LAGs are recorded in the other sections, with only 6 LAGs recorded in the “parallel to the keel” and “transverse to the keel (anterior)” sections.

## 4 Discussion

This study permitted a detailed analysis of the variation in the microanatomy and osteohistology of axial, appendicular and dermal bones of an approximately 14 years old captive, *Crocodylus niloticus*. This study is novel in that it documents the histological variation evident in the skeleton, and in osteoderms from different regions of the body, as well as the variation evident within each osteoderm.

### 4.1 Microanatomy of Axial, Limb, and Dermal Bones

The open MC observed in the appendicular and axial bones in this study, as well as the extensive development of secondary reconstruction in the long bones, and the trabecular (and/or RCs) in medullary regions of the dorsal and cervical ribs are typical of extant and extinct crocodilians (e.g., Audije‐Gil et al. 2023; Mascarenhas‐Junior et al. 2021; Navarro et al. 2023; Pereyra 2021; Waskow and Mateus 2017; Woodward et al. 2014), and are consistent with its semi‐aquatic lifestyle (e.g., Houssaye 2009; de Buffrénil et al. 2015). Similarly, the *diploë* structure of the osteoderms, characterized by two compact cortices (external and basal) enclosing a compact cancellous internal core, has been observed in various pseudosuchians (e.g., Burns et al. 2013; Cajado et al. 2025; Cerda et al. 2013; de Buffrénil et al. 2015; Klein et al. 2019; Ponce et al. 2022; Vickaryous and Hall 2008). The external cortex is usually thicker in the region of the keel, especially in the dorsal and nuchal osteoderms. Although this is related to the porosity in the internal core, the basal cortex in the region of the keel does not show such differences. Previous studies have shown that the osteoderm microstructure and microanatomy can vary depending on their location in the body (Scheyer et al. 2014; Tucker 1997).

### 4.2 Osteohistology of Axial and Appendicular Bones

One of the distinct characteristics of the bone tissues of the appendicular and axial elements is that in the former, changes in the histology were observed throughout ontogeny. The early deposited bone in the appendicular bones consists of WPC or WB interrupted by annuli (sometimes associated with LAGs), whereas in the axial elements the bone formed later is vascularized PFB interrupted by LAGs. On the other hand, the ribs (except for the dorsal‐anterior rib) present PFB interrupted by LAGs throughout the whole compacta. Thus, unlike the appendicular bones, the ribs do not show variation in their growth dynamics since they form a consistent type of bone tissue throughout ontogeny. Although the long bones showed a decrease in the bone depositional rate, it is evident that the animal was still growing at the time of its death. Some studies report that *C. niloticus* reaches sexual maturity at approximately 14 years (e.g., Cott 1961). In our study, the change in the bone tissue type indicates a decrease in the bone depositional rate, which could be related to the attainment of sexual maturity. Such growth dynamics agrees with previous reports in other studies of *C. niloticus* (Hutton 1986), as well as in other modern crocodilians such as *Alligator mississippiensis* (Klein et al. 2009) and *Caiman latirostris* (e.g., Pereyra et al. 2025). In the case of the ribs and gastralia, only the dorsal anterior rib shows substantial histological variation across the cross‐section (i.e., WPC in the ventral area and avascular PFB in the dorsal area). The other ribs and the gastralia generally have the slowest bone depositional rates. Overall, our results confirm that the histology of the appendicular bones of *C. niloticus* is more informative regarding growth dynamics than axial elements.

In general, we found that the limb bones preserved more GMs as compared to the ribs, which suggested that ribs are not ideal for skeletochronology. Here, we found that the radius, ulna, and fibula have 14 GMs, which agrees well with the aproximate age of our crocodile. However, the total number of GMs deposited in the bones of our specimen may have been higher, as GMs formed early in ontogeny were likely lost due to bone resorption/remodeling. Although, previous studies of *Crocodylus siamensis*, *Alligator mississippiensis, Caiman latirostris* showed that the same limb bones maintained a good track record of their growth (Woodward et al. 2014; Pereyra et al. 2024; de Buffrénil 1980).

In our study, the most significant outlier was the gastralia, which preserved approximately 24 GMs. Some of these GMs were closely spaced, forming multiple growth mark clusters (Cullen et al. 2021). Interestingly, a similar arrangement of closely packed GMs was previously reported in the gastralia of a Triassic pseudosuchian (Klein et al. 2017). Perhaps this pattern of extremely slow, interrupted growth with multiple GMs is typical of the growth dynamics of the gastralia.

### 4.3 Osteohistology of the Osteoderms

Here, we describe how the external and basal cortices, and the internal core of the osteoderms varies across our sample, both in terms of location in the body, and the direction of sectioning.

#### 4.3.1 External Cortex of Osteoderms

Ornamentation of the external cortex of osteoderms is a feature widely present in most of extinct and extant crocodilians taxa (e.g., Cerda and Desojo 2011; de Buffrénil et al. 2015; Hua and de Buffrenil 1996; Navarro et al. 2024; Pochat‐Cottilloux et al. 2024; Vickaryous and Hall 2008). The external surface of the outer cortex of the osteoderms from the individual studied here, exhibits a marked ornamentation composed of ridges and valleys. This ornamentation is considerably more pronounced in the nuchal and dorsal osteoderms, while the caudal and flank osteoderms do not exhibit such well‐developed ridges and valleys. Similar results were reported in *Paleosuchus palpebrosus*, in which variation in the development of ornamentation was associated with a greater role of thermoregulation in the dorsal osteoderms (Clarac et al. 2024). It is also interesting to note that although both dorsal and nuchal osteoderms are ornamented, the degree of development of this ornamentation varies among different sectioning planes within each osteoderm. Thus, sectioning planes made “transverse to the keel” and “parallel to the keel” showed a greater presence of ridges and valleys than sections taken “over the keel”. The ornamentation of osteoderms can be formed by two different processes: resorption and redeposition, or by infilling the valleys (de Buffrénil et al. 2015; Vickaryous and Hall 2008). Our data suggest that ornamentation occurs as a result of the former process since we observed the presence of resorption lines followed by the deposition of secondary tissues.

Crocodiles' osteoderms often contain a mixture of PFB, LB and WPC, structural fibers as well as SFs and GMs (e.g., Burns et al. 2013; Cerda and Desojo 2011; Klein et al. 2009; Navarro et al. 2024; Pochat‐Cottilloux et al. 2024; Sena et al. 2023; Vickaryous and Hall 2008). Among the different osteoderms, a broad range of histological types was observed. The flank and tail osteoderms are characterized by a predominance of poorly vascularized PFB, indicating relatively low bone deposition rates. In contrast, the nuchal and dorsal osteoderms exhibit a combination of PFB and WPC associated with a high density of vascularization, reflecting relatively rapid rates of bone deposition. In agreement with the observations of Hua and de Buffrenil (1996), Burns et al. (2013) and Cerda et al. (2018), our results further suggest that the bone tissue organization and vascular density of osteoderms varies widely within a single individual, and that this variation is likely primarily related to osteoderm size and anatomical location.

Sharpey's fibers, responsible for anchoring osteoderms to the surrounding connective tissue (Francillon‐Vieillot et al. 1990; Pochat‐Cottilloux et al. 2024), are commonly reported in the external cortex of osteoderms (e.g., Vickaryous and Hall 2006; Vickaryous and Sire 2009). In most of the osteoderms studied here, there is a notable abundance of SFs associated with the keel, which was observed in both the “transverse to the keel” and “over the keel” sections. This pattern of abundant SFs is similar to the *Sharpey's fibered bone* described by Vickaryous and Hall (2006) and Sun and Chen (2013). Abundant SFs in the keel of osteoderms was also reported in dinosaurs and pseudosuchians (e.g., Pochat‐Cottilloux et al. 2024; Scheyer et al. 2014). Additionally, thick and highly conspicuous SFs are present in the lateral and/or medial ends of the dorsal and nuchal osteoderms, where they are associated with the articulation between adjacent osteoderms (e.g., Cerda and Desojo 2011; Hill 2010; Klein et al. 2009).

#### 4.3.2 Internal Core of the Osteoderms

A highly porous internal core has been observed in several crocodilians (de Buffrénil et al. 2015). The reconstruction of the internal core has been linked to differences in the sex of the crocodiles, as females show more extensive reconstruction than males, likely reflecting the mobilization of calcium to support eggshell calcification during reproduction (Klein et al. 2009; Tucker 1997). It may also be related to ontogeny, since the porosity of the internal core tends to increase with age (Cerda et al. 2013). The internal core of the osteoderms of the crocodile under investigation here (Cott 1961) is highly porous (cancellous) and has CCCB indicating high rates of remodeling, which are all consistent with its estimated age and likely sexually mature status (Cott 1961). (e.g., Dacke et al. 2015; Tucker 1997; Wink et al. 1987)

In our study we found that the development of porosity and thickness of the internal core is highly variable among the different osteoderms. The lateral nuchal, flank, dorsal‐posterior, and tail osteoderms exhibit a proportionally extensive and highly porous internal core. Moreover, analyses within individual osteoderms revealed that the keel region consistently contains markedly larger resorption spaces than the other regions of the osteoderms. This suggests higher remodeling and reconstruction rates in the region of the keel which could be associated to its formation and ossification process. Indeed, Vickaryous and Hall (2008) reported that in *A. mississippiensis*, the keel is the first region to ossify and that thereafter, the ossification is centrifugal. Some extant and extinct crocodilians show similar features in the internal core in the region of the keel (e.g., Burns et al. 2013).

#### 4.3.3 Basal Cortex of Osteoderms

The basal cortex is predominantly composed of vascularized PFB interspersed with numerous GMs, as reported for several crocodilians (e.g., de Buffrénil et al. 2015). Despite the marked histological variation identified in other regions of the osteoderms, the basal cortex appears to be the most consistent since no significant differences were observed within individual osteoderms across the different sectioning planes. In most osteoderms, vascularization is more abundant in the deeper portions of the basal cortex and progressively decreases toward the outer surface.

Sharpey's fibers are generally poorly represented in the basal cortex of most sections, although the nuchal, dorsal anterior and dorsal posterior osteoderms show high abundance. In these osteoderms, the sections “over the keel” show conspicuous SFs in the posterior area. This organization concurs with previous studies (e.g., de Buffrénil et al. 2015), which describe SFs in the basal cortex as anchoring structures for connective tissues particularly in regions subjected to high mechanical stress i.e., dorsal region (Cerda et al. 2014; Dubansky et al. 2026; Vickaryous and Hall 2008). This structural adaptation enhances mechanical strength and provides a stable base for the attachment of connective tissues in high‐load areas such as the neck and back. In our study, we found a lower density of SFs in the basal cortex in comparison with the external cortex. This distribution pattern is consistent with observations in notosuchians (Cajado et al. 2025).

#### 4.3.4 Growth Marks of Osteoderms

Our data revealed that the number of GMs varied substantially depending on the location of the osteoderm and on the plane of sectioning (Taborda et al. 2013)

The dorsal anterior osteoderm preserved the highest number of GMs in most of the planes of sections: 16 GMs “over the keel”, 14 GMs “parallel to the keel”, and 14 GMs “transverse to the keel (posterior)”. In contrast, the flank, and tail osteoderms retained far fewer GMs (i.e., between 6 and 10 in the flank osteoderm, and between 3 and 9 in the tail osteoderm). The nuchal osteoderm had between 8 and 12 GMs, and the nuchal lateral osteoderm had between 8 and 13 GMs. We also found that the external cortex preserves more GMs than the basal cortex. Contrary to our results, Hutton (1986) found that the longitudinal section just near the keel in the nuchal osteoderm recorded the highest number of GMs (which he proposed was the best for age estimation in *C. niloticus*), whereas, Tucker (1997) found that the transverse section of the nuchal osteoderm in *C. johnstoni* was best for skeletochronology.

Our study indicates that the lateral nuchal and anterior dorsal osteoderms preserve the number of GMs most closely matching the likely age of the individual (14 years old), although the anterior dorsal osteoderm exhibits a slight excess (16 GMs). However, it is important to consider that osteoderm ossification begins after the first year of life, with ossification initiating in the nuchal region and subsequently progressing posteriorly along the body (Vickaryous and Hall 2008). Consequently, our results suggest that the number of GMs recorded in the lateral nuchal and anterior dorsal osteoderms exceeds the expected number for our individual, suggesting the presence of stochastic growth marks in these osteoderms, leading to an overestimation of the animal's age (Chinsamy and Pereyra 2026). Although previous studies on extant crocodilians (Woodward et al. 2014) have suggested that osteoderms generally preserve fewer GMs than expected, our results show that osteoderms can sometimes preserve a higher number of GMs.

## 5 Conclusion

This study demonstrates intraskeletal variation in the histology of axial and appendicular skeletal elements, as well as among osteoderms from different parts of the dermal skeleton of an approximately 14‐year‐old *Crocodylus niloticus*. We found substantial variation in the number of GMs preserved in the postcranial bones with the fibula, ulna, and radius showing GMs commensurate with the age of this individual. Interestingly, although the gastralia records the highest number of GMs, it over‐estimates the age of the crocodile, and emphasizes the fact that GMs can sometimes form in clusters (e.g., doublets or triplets), that are challenging to discern. With regard to the osteoderms, the dorsal anterior osteoderm preserved the highest number of GMs, which also exceeds the age of the individual. Furthermore, not all osteoderms from a single individual will necessarily share the same histology. Although previous studies on extant crocodilians (e.g., Woodward et al. 2014; Audije‐Gil 2023) have suggested that osteoderms generally preserve fewer GMs than expected (because of extensive secondary remodeling), our results show that osteoderms can sometimes preserve GMs exceeding the age of the individual.

## Author Contributions

**Ayanda Buso:** wrote first draft which formed part of her BSc. (Hons) thesis, implementation of methods, visualization. **Maria Eugenia Pereyra:** conceptualization, investigation, visualization writing – review and editing, validation, formal analysis, project administration, data curation, supervision. **Anusuya Chinsamy:** conceptualization, investigation, writing – review and editing, methodology, project administration, supervision.

## Supporting information


Supporting File 1

Supporting File 2

## Data Availability

Journal of Zoology (JZO).
