# Supplementary material for: Biological Insights From the Osteohistology of a Nile Crocodile
Source: J Morphol. 2026 Oct 26;287(10):e70186. doi: 10.1002/jmor.70186 (PMC13700083; doi:10.1002/jmor.70186)
Supplement: Supplementary file 1 [file JMOR-287-e70186-s002.docx]

Supplementary information:

Table S1. Measurements of appendicular bones of *Crocodylus niloticus* specimen under study.

| Bone | Length (mm) | Distal end (mm) | Proximal end (mm) | Medial lateral diaphysis (mm) | Anterior posterior diaphysis (mm) |
| --- | --- | --- | --- | --- | --- |
| Femur | 201.35 | 54.54 | 44.81 | 24.39 | 27.23 |
| Humerus | 179.09 | 54.67 | 53.26 | 22.94 | 25.09 |
| Tibia | 142.96 | 42.77 | 49.57 | 21.17 | 17.63 |
| Fibula | 138.34 | 21.46 | 24.84 | 10.29 | 10.94 |
| Ulna | 122.51 | 25.56 | 40.08 | 21.34 | 12.32 |
| Radius | 104.8 | 31.92 | 31.47 | 13.18 | 15.08 |

Table S2. Measurements of axial bones of *Crocodylus niloticus* specimen under study.

| **Ribs** | Length (mm) | Distal end (mm) | Proximal end (mm) | Dorsoventral diaphysis (mm) | Anterior posterior diaphysis (mm) |
| --- | --- | --- | --- | --- | --- |
| Cervical | 55.05 | 5.5 | 26.6 | 5.43 | 13.9 |
| Anterior dorsal | 141.87 | 11.02 | 56.69 | 8.15 | 14.99 |
| Posterior dorsal | 182.63 | 14.24 | 23.83 | 5.44 | 12.89 |
| gastralia | 148.87 | 5.38 | 6.84 | 3.32 | 5.34 |

Table S3. Measurements of osteoderms of *Crocodylus niloticus* specimen under study.

| Osteoderms | Anterior posterior (mm) | Medial lateral (mm) | Thickness (mm) |
| --- | --- | --- | --- |
| Nuchal | 62.54 | 83.57 | 19.53 |
| Nuchal lateral | 58.09 | 49.62 | 19.97 |
| Dorsal anterior | 51.7 | 76.65 | 15.09 |
| Dorsal posterior | 49.55 | 58.05 | 14.32 |
| Flank | 41.74 | 28.12 | 9.25 |
| Tail | 35.95 | 23.54 | 11.33 |

Figure S1. Axial and appendicular bones of *Crocodylus niloticus* and their corresponding CT Scan images of the region where the thin sections were performed. The red lines represent where the bones were cut for thin sectioning. The white lines are the 2mm scale. The blue lines are the scales of the CT-scan. A, humerus. B, radius. C, ulna. D, femur. E, fibula. F, tibia. G, gastralia. H, anterior-dorsal rib. I, cervical rib. J, posterior-dorsal rib.

Figure S2. Osteoderms of *Crocodylus niloticus* studied and their corresponding CT Scan images of the thin sections of the region where the thin sections were performed. The red lines represent where the bones were cut for thin sectioning. A, nuchal osteoderm. B, over the crest thin section of A. C, parallel to the crest thin section of A. D, transversal anterior thin section of A. E, transversal posterior thin section of A. F, nuchal-lateral osteoderm. G, over the crest thin section of F. H, parallel to the crest thin section of F. I, transversal thin section of F. J, dorsal anterior osteoderm. K, over the crest thin section of J. L, parallel to the crest thin section of J. M, transversal anterior thin section of J. N, transversal posterior thin section of J. O, dorsal posterior osteoderm. P, over the crest thin section of O. Q, parallel to the crest thin section of O. R, transversal anterior thin section of O. S, transversal posterior thin section of O.

Figure S3. Osteoderms of *Crocodylus niloticus* and their corresponding CT Scan images of the thin sections of the region where the thin sections were performed. The red lines represent where the bones were cut for thin sectioning. A, flank osteoderm. B, over the crest thin section of A. C, parallel to the crest thin section of A. D, transversal anterior thin section of A. E, transversal posterior thin section of A. F, tail osteoderm. G, over the crest thin section of F. H, parallel to the crest thin section of G. I, transversal anterior thin section of G. J, transversal posterior thin section of G.
